# Origin-Independent Circular Dichroism Spectra for Molecular and Hybrid Systems with Real-Time Time-Dependent Density Functional Theory

**DOI:** 10.1021/acs.jpca.6c03456

**Published:** 2026-10-20

**Authors:** Maria J. Weseloh, Mikael Kuisma, Xi Chen, Patrick Rinke

**Affiliations:** † Department of Applied Physics, 174277 Aalto University, P.O. Box 11100, FI-00076 AALTO Espoo, Uusimaa, Finland; ‡ CAMD, Department of Physics, 5205 Technical University of Denmark, Fysikvej 309, 2800 Kgs. Lyngby, Denmark; § Lanzhou Center for Theoretical Physics, Lanzhou University, Gezhi Building and Science and Engineering Building, No. 222, Tianshui South Road, Lanzhou 730000, Gansu, China; ∥ Physics Department, TUM School of Natural Sciences, Technical University of Munich, 85748 Garching, Germany; ⊥ Atomistic Modelling Center, Munich Data Science Institute, Technical University of Munich, Walther-Von-Dyck Str. 10, 85748 Garching, Germany; # Munich Center for Machine Learning (MCML), 80804 Munich, Germany

## Abstract

We present a velocity gauge implementation for real-time time-dependent density functional theory (RT-TDDFT) calculations of electronic circular dichroism (ECD) spectra within the linear combination of atomic orbitals framework of the GPAW code. In contrast to the commonly used length gauge, which exhibits a pronounced dependence on the origin of the coordinate system, our velocity gauge implementation produces origin-independent ECD spectra already for moderately sized basis sets. The computational efficiency of the RT-TDDFT implementation in GPAW enables efficient ECD simulations of large finite systems, making chiroptical analyses of molecular and molecule–nanoparticle assemblies exceeding 1000 atoms feasible.

## Graphical abstract

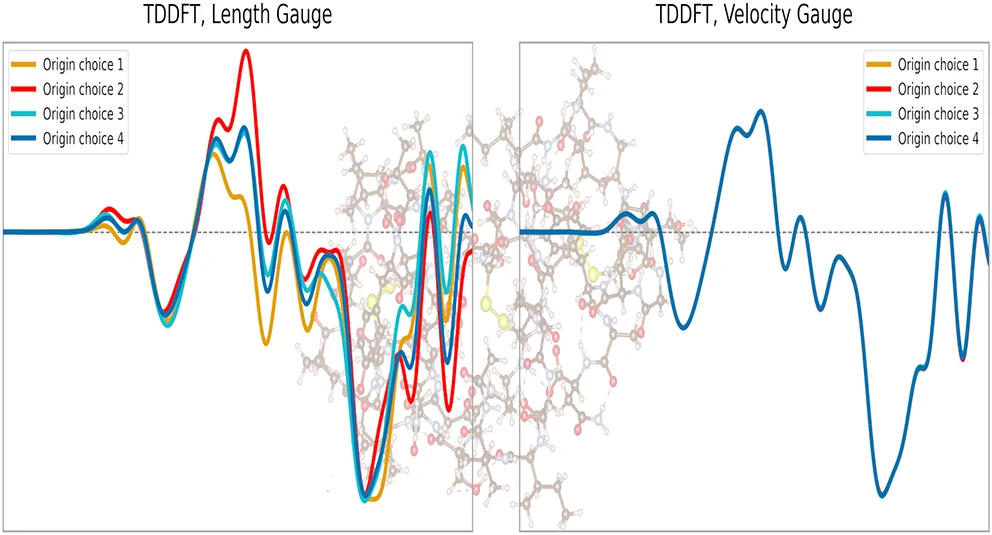

## Introduction

Many enzymes and receptors in nature are intrinsically chiral, and thus bind selectively to molecules according to their handedness. This chiral dependence can lead to markedly different effects of enantiomers on humans and in nature.^4,12^ Consequently, the accurate determination of molecular chirality is of critical importance in numerous research fields, including drug safety and environmental monitoring.^39,53^

Among available methods, ECD spectroscopy has emerged as a powerful probe of chirality owing to its exceptional sensitivity to stereochemical configuration.^5^ Its broader applicability, however, is limited because many molecules generate ECD signals below the detection threshold; hence, strategies to amplify these responses are highly desirable. Already in the early 2010s, it was recognized that molecular chiral signals in ECD spectra can be enhanced through interactions with achiral plasmonic nanoparticles.^33,48^

In addition, interpreting ECD spectra on the basis of experiments alone can be challenging, as the correspondence between spectral features and molecular structure is often indirect and nontrivial.^14^ To obtain deeper insight, theoretical simulations have become indispensable: computational methods establish a direct link between molecular geometry and the observed ECD response, thereby providing a powerful tool for analysis.^49,55^ In principle, a comprehensive analysis of both intrinsic ECD and plasmonic enhanced ECD spectra, without reliance on experimental input parameters, can be achieved using TDDFT-based ECD calculations.^31,49^ However, many such response-calculations exhibit a pronounced dependence on the choice of coordinate-system origin, see for example ref 26, Results section, and Figure 8 of Appendix A in ref 20. The latter shows the energy-dependent divergence of ECD spectra from different origins and also shows that the divergence is enhanced in a spatially more extended hybrid system due to a larger nanoparticle, compared to the same molecule combined with a smaller nanoparticle. The origin dependence is closely tied to the gauge adopted in the TDDFT implementation. In particular, the common length gauge is prone to such an origin dependence.^34^

TDDFT, however, remains the ECD method of choice since it favorably balances accuracy and computational cost. Hence, it has found widespread application across various fields,^52^ including spectroscopy^6^ and nanoplasmonics.^44^ TDDFT is available in two main formulations: linear-response TDDFT (LR-TDDFT) and real-time TDDFT (RT-TDDFT). LR-TDDFT is a frequency-domain approach that employs a perturbative expansion of the time-dependent Kohn–Sham (KS) equations,^11,45^ and is commonly used to compute excitation energies and oscillator strengths. In contrast, RT-TDDFT directly propagates the time-dependent KS equations in real time,^57^ allowing for the simulation of ultrafast electron dynamics and nonlinear responses.^29^ While LR-TDDFT often becomes computationally prohibitive for systems with several hundred atoms due to its implementation-dependent scaling between $O\left(N^{3}\right)$ and $O\left(N^{6}\right)$ with system size *N*,^8,51^ RT-TDDFT, despite its larger prefactor, usually offers more favorable scaling and is thus better suited for calculations on larger systems.^51^ A common approach to mitigate the gauge-origin dependence of length-gauge TDDFT calculations is the use of London atomic orbitals (LAOs), also known as gauge-including atomic orbitals (GIAOs).^26,40,56^ Such orbitals augment Gaussian basis functions with a magnetic-field-dependent phase factor that depends on the position of the nucleus relative to the gauge origin.^26,46^ For velocity-gauge calculations using such basis sets origin independence can be harder to achieve due to slower basis-set convergence.^40^ Although LAOs/GIAOs mitigate the gauge-origin dependence of the length-gauge formulation, this advantage comes at the cost of complex, magnetic-field-dependent basis functions and more elaborate integral evaluations^46^ compared with conventional LCAO basis sets, which can increase the computational overhead for large systems.

To overcome the origin dependence while retaining computational efficiency, we implemented the velocity gauge in GPAW’s projector augmented-wave (PAW) RT-TDDFT framework^7^ with its linear combination of atomic orbitals (LCAO) basis sets.^28^ The GPAW-LCAO RT-TDDFT implementation considered here scales as $O\left(N^{3}\right)$ . In contrast, the corresponding GPAW LR-TDDFT formulation requires $O\left(N^{5}\right)$ for constructing the response matrix. Within the GPAW LR-TDDFT formalism the number of electron–hole pairs scales as $O\left(N^{2}\right)$ , resulting in $O\left(N^{4}\right)$ response-matrix elements, each requiring an O(N) real-space integral and the full diagonalization of the response matrix scales as $O\left(N^{6}\right)$ . Consequently, the RT-TDDFT approach offers a markedly more favorable asymptotic scaling for the large systems investigated in this work. However, while velocity-gauge origin independence has also been realized in pseudopotential (PP) methods, as, for example, in ref 34, our contribution is a new PAW–LCAO-based implementation whose computational efficiency enables RT-TDDFT ECD for hybrid systems and large molecules. PP methods offer another route to computational efficiency. The PAW approach and the PP approach differ fundamentally in their treatment of the core region. In the PAW method, smooth pseudo wave functions are transformed back to all-electron wave functions by adding atom-centered on-site corrections; thus all-electron quantities can be reconstructed within the frozen-core approximation.^7,25^ In PP methods, the core electrons and the rapidly varying all-electron wave functions in the core region are replaced by an effective, usually nonlocal potential, such that only pseudo valence states are treated explicitly.^2,24,25^ The absence of an explicit all-electron reconstruction in PP methods can affect the calculation of magnetic dipole moments. Consequently, ECD calculations using PPs require additional corrections to the magnetic dipole moment operator, as for example introduced in ref 34. We further investigate whether the velocity gauge can effectively eliminate origin dependence in hybrid systems involving plasmonic nanoparticles. In addition, we assess the basis-set dependence of ECD spectra within the LCAO RT-TDDFT framework. If moderately sized basis sets in the velocity gauge yield sufficiently converged and origin-independent ECD spectra, this would pave the way for large-scale investigations of chirality.

## Theory and Implementation

### ECD RT-TDDFT Formalism in GPAW

In the LCAO mode of GPAW, the KS pseudowave functions are represented as linear combinations of localized atomic-like basis functions. The wave functions^28^ are expressed as

1 $${\mathrm{\psi ̃}}_{nk}\left(r\right)=\sum_{\mu }c_{\mu }^{nk}\underset{{\mathrm{\Phi ̃}}_{k\mu }\left(r\right)}{\underset{︸}{\sum_{R}e^{ik\cdot R}{\mathrm{\Phi ̃}}_{\mu }\left(r-R\right)}}$$

where ψ̃_*n* **k**_(**r**) denotes the pseudowave function for band index *n* and wave vector **k**, and Φ̃_μ_(**r**) are the atomic-like basis functions indexed by μ. μ is a compound index for the radial and spherical harmonic parameters (*n, l, m*) and the atomic index *a*.

Each basis function is constructed as the product of a numerical radial function and a spherical harmonic

2 $${\mathrm{\Phi ̃}}_{\mu }\left(r\right)={\mathrm{\varphi ̃}}_{\mathrm{nl}}\left(r^{a}\right)Y_{\mathrm{lm}}\left({\mathrm{r̂}}^{a}\right)$$

where *r* ^*a*^ = ∥**r** – **R** ^*a*^∥ is the distance from the nucleus at position **R̂** ^**a**^, and **r̂** ^**a**^ is the unit vector of **r** – **R** ^*a*^. φ̃_*nl*_(*r*) is a tabulated radial function, and *Y*_*lm*_(**r̂**) a spherical harmonic. More details can be found in refs 28 and 38.

Due to the diverging Coulomb potential, electronic wave functions exhibit rapid oscillations near the atomic nuclei. As mentioned, in the GPAW code numerical problems from these oscillations are counteracted by using the PAW method.^7^ In the PAW formalism, the computationally demanding wave functions near the core are replaced by smooth auxiliary wave functions, while the wave functions farther away from the core, outside the augmentation sphere,^38^ coincide with the all-electron wave functions. More details can be found in the publication about LCAO basis sets in the PAW formalism ref 28.

### Implementation of the LCAO-Velocity Gauge

### Gauge Transformation

We introduce the electric field **E** that probes ECD in terms of a vector **A** and a scalar Φ potential, $E=-\nabla \Phi -\frac{\partial A}{\partial t}$ . These potentials are not directly measurable and different representations of the electromagnetic potentials can produce the same physically observable electric field. Different representations are related by a gauge transformation

3 $$\begin{array}{l} A\to A^{\prime }=A+\nabla \chi \left(r,t\right)\\ \Phi \to \Phi^{\prime }=\Phi -\frac{\partial \chi \left(r,t\right)}{\partial t} \end{array}$$

where χ is a scalar function. Although the electric field and other observables should, in principle, be unchanged by gauge transformations, practical calculations can sometimes produce gauge-dependent results. This discrepancy arises from numerical approximations, finite basis sets, and limitations of the exchange-correlation functional, which can break gauge invariance at the computational level.^16,18^

Two common gauges are the length and the velocity gauge. They are related by the gauge transformation χ­(**r**,*t*) = **r**·**A**(*t*). The length gauge is obtained for **A**′(*t*) = **0**, where the light–matter interaction term in the Hamiltonian exhibits an explicit spatial dependence

4 $${Ĥ}_{\mathrm{int}}^{\mathrm{LG}}=r\cdot E\left(t\right)$$

This spatial dependence facilitates the appearance of the origin dependence in ECD spectra.

In the velocity gauge we have Φ′(**r**,*t*) = 0, and the light–matter interaction term does not introduce any explicit spatial dependence in the Hamiltonian

5 $${Ĥ}_{\mathrm{int}}^{\mathrm{VG}}=A\left(t\right)\cdot \mathrm{p̂}$$

where **p̂** is the momentum operator. This interaction Hamiltonian in the velocity gauge is obtained from the minimal-coupling Hamiltonian in the long-wavelength or dipole approximation, where the vector potential is spatially homogeneous, **A**(**r**,*t*) → **A**(*t*). In this limit, the diamagnetic term *q* ^2^ **A** ^2^(*t*)/(2*m*) is independent of **r**. The diamangetic term can be eliminated by a time-dependent unitary transformation of the wave function, which adds a spatially uniform phase and leaves all observables unchanged. After this phase transformation, the velocity-gauge interaction Hamiltonian can be written as in eq 5. This corresponds to a time-dependent shift of the energy zero and is the standard form used in the dipole approximation. Since the vector potential **A**(*t*) does not depend on **r,** the magnetic field is zero, according to the Maxwell equations. In case of a perturbing external magnetic field with a position-dependent vector potential, the transformation of the pseudowave functions may lead to origin-dependent terms and additional PAW-corrections are generally required, as discussed in ref 42. Since no magnetic-field kick is used here, these corrections are not relevant for the present calculations. While we treat the external potential in the velocity gauge, we treat the underlying ground-state calculation fully in the Coulomb gauge.

To simulate the system’s response to a time-dependent electromagnetic pulse, we model the applied vector potential as

6 $$A\left(t\right)=\Theta \left(t\right)A_{\mathrm{ext}}$$

Here, Θ­(*t*) is the Heaviside step function and **A**_ext_ = –κ**ê**_*i*_ the applied vector potential, where κ is its amplitude, respective the integrated intensity of the applied δ-kick, and **ê**_*i*_ is the unit vector to a particular direction. This choice of vector potential corresponds to the application of a constant electric field in form of a delta-pulse kick

$$E=-\frac{d}{dt}\left\{\Theta \left(t\right)A_{\mathrm{ext}}\right\}=\delta \left(t\right)\kappa {ê}_{i}$$ 7

where δ­(*t*) is the Dirac delta function and **A**_ext_ is the constant vector potential that is switched on at time *t* = 0^+^. Since the kick is represented by a Heaviside step function, the time propagation starts from the ground-state KS orbitals obtained for the unperturbed system. At *t* = 0^+^, the external vector potential is switched on, which modifies the Hamiltonian by adding the term in eq 5. This sudden change means that the previously computed ground state is no longer an eigenstate of the new Hamiltonian. As a result, the system is driven out of equilibrium and begins to evolve in time under the influence of the altered potential.

### Calculation of ECD Spectra

For the calculation of ECD spectra, we evaluate the expectation value of the magnetic dipole moment operator^31^

8 $$M=\sum_{n}f_{n}\left\langle \Psi_{n}\right|r\times \mathrm{p̂}\left|\Psi_{n}\right\rangle$$

where *f*_*n*_ are the occupation numbers and *n* runs over all states. Equation 8 remains unchanged from the implementation published in ref 31 and is used for calculations performed in length and velocity gauge.

The experimentally measured quantity in ECD is the differential absorption of left- and right-circularly polarized light. Its theoretical counterpart is the rotatory strength *R*(ω), which characterizes the ECD response at energy ω. In this article, we use the term ECD to refer to the rotatory strength *R*(ω). The expression for *R*(ω) is^31^

9 R(ω)=1πκRe[∑kmk(k)(ω)]

Here κ = |∫_–∞_^∞^ **E** d*t*| denotes the integrated intensity of the applied δ-kick, and *m*_*k*_^(*k*)^(ω) is the Fourier-transformed magnetic dipole moment induced by a kick in direction (*k*). *k* runs over the Cartesian directions *x*, *y*, *z*.

The δ-kick drives the system’s dynamic response. The magnetic dipole moment responds to the kick’s perturbation: its components oscillate as the system propagates under the new Hamiltonian, and these oscillations encode the chiral response. The Fourier transform of this time-dependent magnetic dipole moment provides *m*_*k*_^(*k*)^(ω), which enters eq 9 to yield the rotatory strength *R*(ω). Because the evaluation of the induced magnetic dipole moment can exhibit origin dependence to varying degrees in length and velocity gauge, the resulting rotatory strength may differ between the two gauges.

In line with previous observations,^35^ subtracting a reference from the magnetic-dipole signal can introduce a spurious peak at zero frequency. Concretely, one computes *m*′(*t*) = *m*(*t*) – *m*(*t*_0_); any small error in the reference *m*(*t*_0_) (e.g., due to numerical noise) produces a constant offset. The Fourier transform of a constant is proportional to a Dirac delta at ω = 0, yielding the artificial zero-frequency peak. This artifact can be removed by estimating and subtracting the correct DC offset of *m*(*t*), for example by integrating *m*(*t*) against the transform’s window/envelope function.

Our implementation is available in the development version of GPAW.

### Computational Details

All calculations presented in the Results section were performed with a development version of the GPAW code, mainly using LCAO basis sets at the augmented double-ζ polarization (aug-dzp) or double-ζ polarization (dzp) level, apart from the basis-set quality dependent calculations. The aug-dzp basis sets were generated from dzp basis sets by adding diffuse augmentation functions, as described in the Supporting Information (SI). For the nanoparticles in the hybrid system we used p-valence basis sets, whereas we used aug-dzp basis sets for the molecules in the hybrid systems. The p-valence basis sets are similar to standard dzp basis sets but include additional p-type valence functions representing atom-centered unoccupied p-states with double-ζ representation. The p-valence basis sets correspond to the basis sets used in ref 27. We used the PBE generalized-gradient-approximation exchange-correlation functional.^41^

For LCAO basis-sets-independent reference results, we additionally conducted calculations with GPAW’s real-space grid method. We used a grid spacing parameter of 0.15 Å to expand the wave functions in real space.

In the LCAO ground-state calculations, a grid spacing of 0.11 Å was used to represent the electron density and the potentials. This corresponds to a real-space grid parameter of *h* = 0.22 Å, noting that densities and potentials are represented on a double-grid scheme. We used a density convergence criterion of 10^–12^.

All systems were embedded in a nonperiodic simulation box with a vacuum of at least 9 Å on each side. To mitigate artifacts from imposing a zero Dirichlet boundary on the Hartree potential, we computed the potential on an enlarged (padded) grid extending beyond the original simulation domain by using GPAW’s ExtraVacuumPoissonSolver. This provides stability for electrostatics of large systems beyond the usually used multipole corrections.

For the length gauge, the electric perturbation was introduced as a delta-kick at *t* = 0 using GPAW’s RT-TDDFT absorption-kick formalism. In the velocity-gauge picture, the corresponding electric field is represented by a step-like change of the external vector potential. Thus, there is no change in the wave functions at time *t* = 0, but the Hamiltonian will be different from time *t* = 0. The subsequent real-time propagation then starts from this perturbed initial state (perturbed from the perspective of the new introduced vector potential which is used for the propagation); no finite-width box-shaped electric-field pulse is applied during the propagation. RT-TDDFT calculations were performed with a kick-strength corresponding to 2.72 eV. A time step of 10 attoseconds over a total propagation time of 30 fs was used, which corresponds to a spectral resolution of 0.14 eV. The resulting ECD spectra were Gaussian-broadened prior Fourier transform with $f\left(t\right)=\exp\left(-\frac{1}{2}\sigma_{w}^{2}t^{2}\right)$ and using the width σ_*w*_ = 0.2 eV.

To test our implementation, we calculated and compared ECD spectra in length and velocity gauges. To assess origin dependence in both gauges, the coordinate origin was systematically shifted to 27 different positions within a cube of 20 Å side length. The corresponding coordinates can be found in Table S1.

Methyl-oxirane adsorbed on an icosahedral 923-atom gold nanoparticle (Au_923_) is the largest hybrid system considered in this work. The calculations for this system were performed on the CSC high-performance computing cluster Mahti using the medium partition. The RT-TDDFT calculations were run on 8 nodes, corresponding to a total of 512 CPU cores (64 cores per node). We did not test the performance of the TDDFT calculations for this system with other numbers of nodes.

For the molecule crambin, we employed a dzp basis set belonging to a large orbital space consisting of 5826 orbitals. Before performing the DFT and TDDFT calculations, we generated a DFT-ready xyz structure by retrieving the 1CRN PDB entry from https://www.rcsb.org/structure/1CRN and processing it with AmberTools25 ^9,10^ for system setup, conjugate-gradient relaxation, and format conversion. Apart from including van der Waals interactions via Grimme’s DFT-D3 correction,^17^ the computational settings were identical to those used for the smaller molecules discussed in this work.

### Studied Systems

We computed spectra for four representative small molecules, comprising the epoxide methyl-oxirane, the nucleobases adenine and cytosine, and the amino acid aspartic acid. The molecules are shown in Figure 1. Methyl-oxirane serves as a widely used benchmark system in chiroptical spectroscopy (see, e.g., ref 47). It is to note that isolated, idealized planar adenine and cytosine are achiral, as the molecular plane constitutes a mirror plane. Nonetheless, multiple low-energy nonplanar conformers are well established,^22,54^ and interactions with surfaces or within host environments can remove the mirror plane, yielding chiral conformers, as reported for adenine.^13,32^ This is in congruence with our calculations for adenine and cytosine on nanoparticles. Since the presented implementations were developed to obtain reliable ECD spectra for hybrid systems, we use the nucleobases’ chiral conformers on the nanoparticle. We show the ECD spectra of these same conformers for the isolated nucleobases since they were already available from enhancement analyses and also contain relevant information for the present study. We have omitted the presentation of adenine-on-nanoparticle spectra, as the ECD of hybrid systems is not the central focus of the present work.

**Figure 1:**
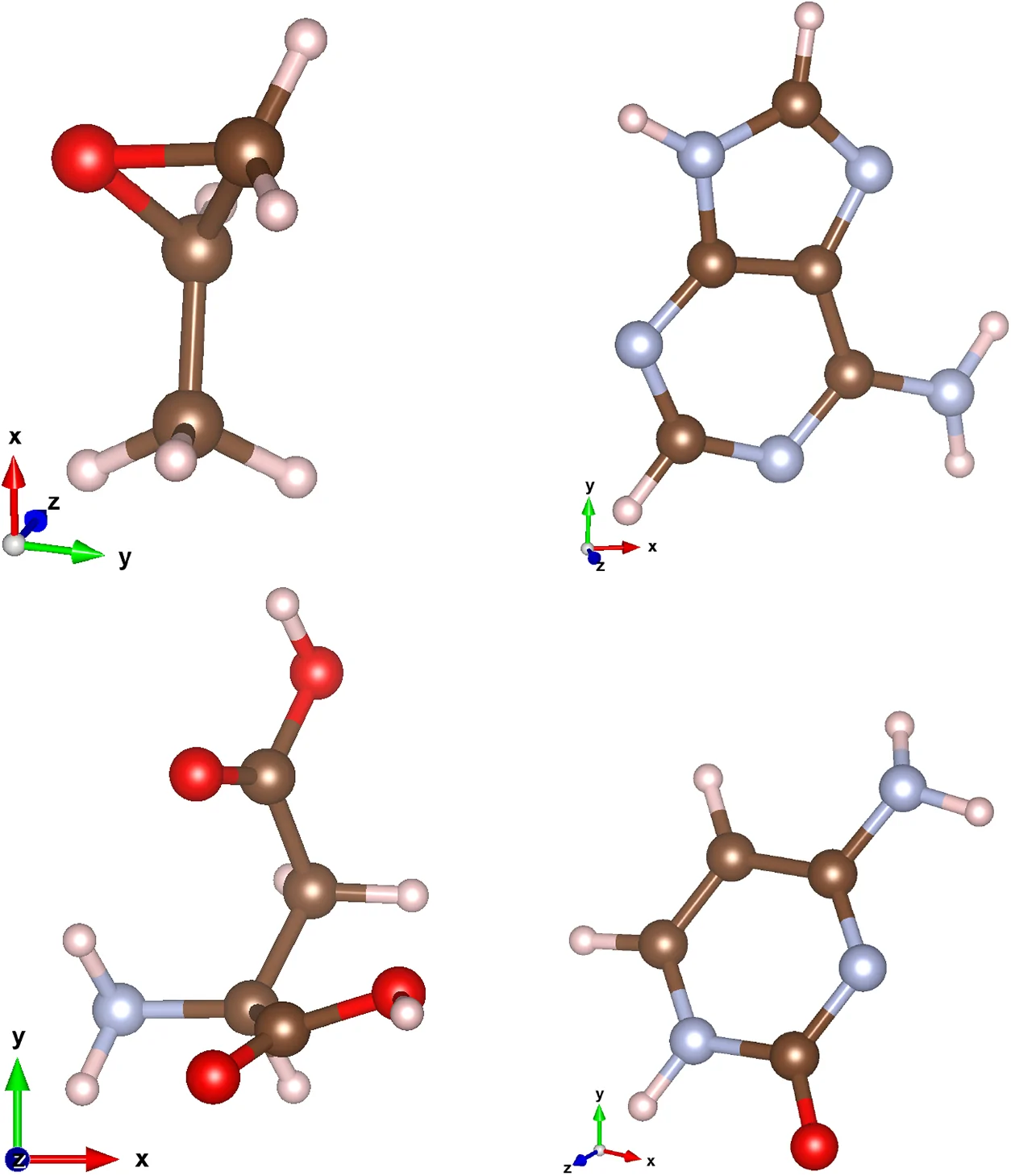
Atomic structures of methyl-oxirane, adenine, aspartic and cytosine (from the upper left to the lower right). Carbon atoms are shwon in brown, hydrogen atoms are shown in pink, nitrogen atoms are shown in light-blue, and oxygen atoms are shown in red. The plots were created using VESTA version 3.5.7.^36^

Due to the high computational cost of TDDFT, ECD calculations are typically restricted to relatively small molecular systems. Simulations for molecules comprising several hundred to a thousand atoms remain challenging.^3^ Consequently, routine applications to larger organic molecules are uncommon, and fragment-based or simplified TDDFT approaches have emerged.^1,3^ This motivated us to test our implementation on crambin (shown in Figure 2), a chiral protein consisting of 642 atoms. In addition, we considered the hybrid systems shown in Figure 3: cytosine adsorbed on Ag_147_ and methyl-oxirane adsorbed on Au_923_.

**Figure 2:**
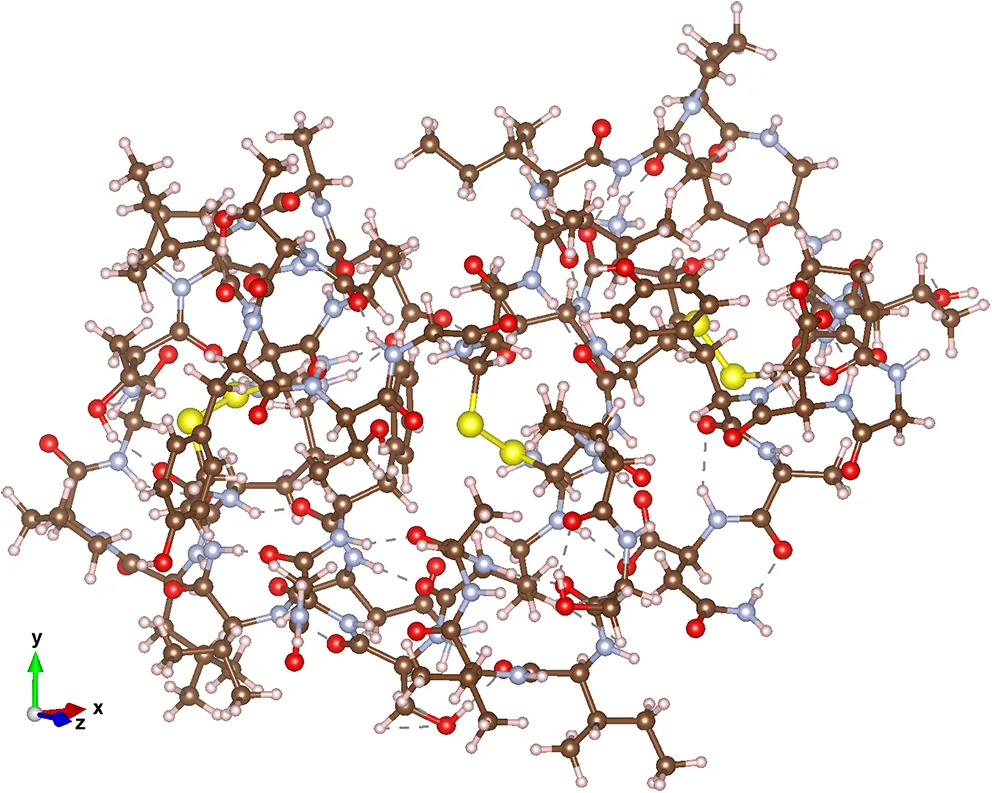
Atomic structure of crambin. Carbon atoms are shown in brown, hydrogen atoms are shown in pink, nitrogen atoms are shown in light-blue, oxygen atoms are shown on red, and sulfur atoms are shown in yellow. The plot was created using VESTA version 3.5.7.^36^

**Figure 3:**
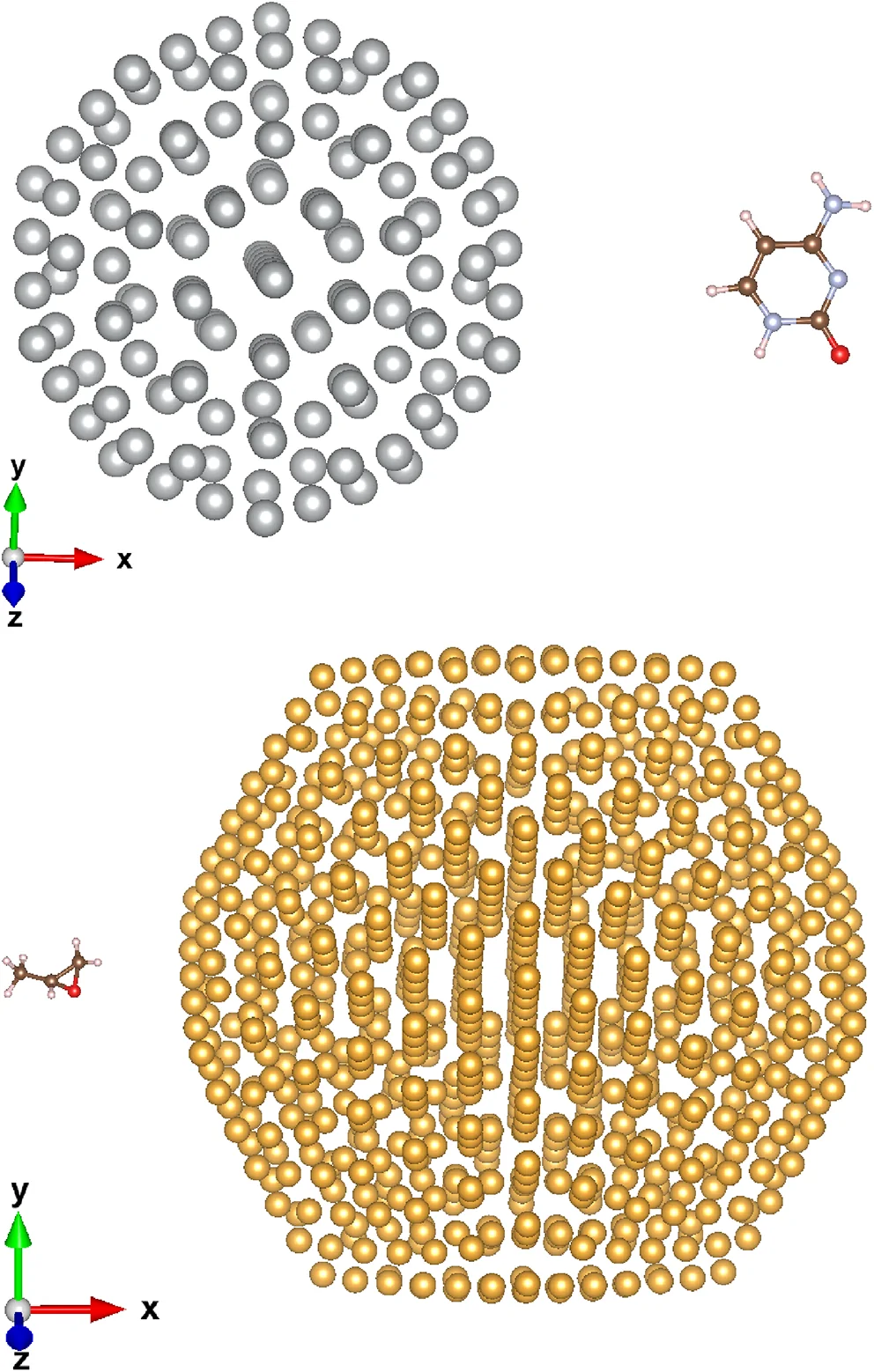
Spatial arrangement of the hybrid systems. Upper part: Cytosine adsorbed on an icosahedral silver nanoparticle containing 147 atoms (Ag_147_). Lower part: Methyl-oxirane adsorbed on Au_923_. The plot was created using VESTA version 3.5.7.^36^

## Results

Figure 4 compares the ECD spectra of methyl-oxirane, adenine, and aspartic acid, calculated within the length (left column) and the velocity gauge (right column) for the aug-dzp basis sets. The spectra were computed for 27 origins under identical computational settings, with the only difference being the used gauge. To illustrate the problem of origin dependence, we show ECD spectra at energies beyond the ionization limit, although LCAO basis sets are known to exhibit deficiencies at energies above the ionization limit.^15,37^ We especially show methyl-oxirane’s ECD spectra above the ionization limit, which is expected to lie in the vicinity of 10 eV.^43^

**Figure 4:**
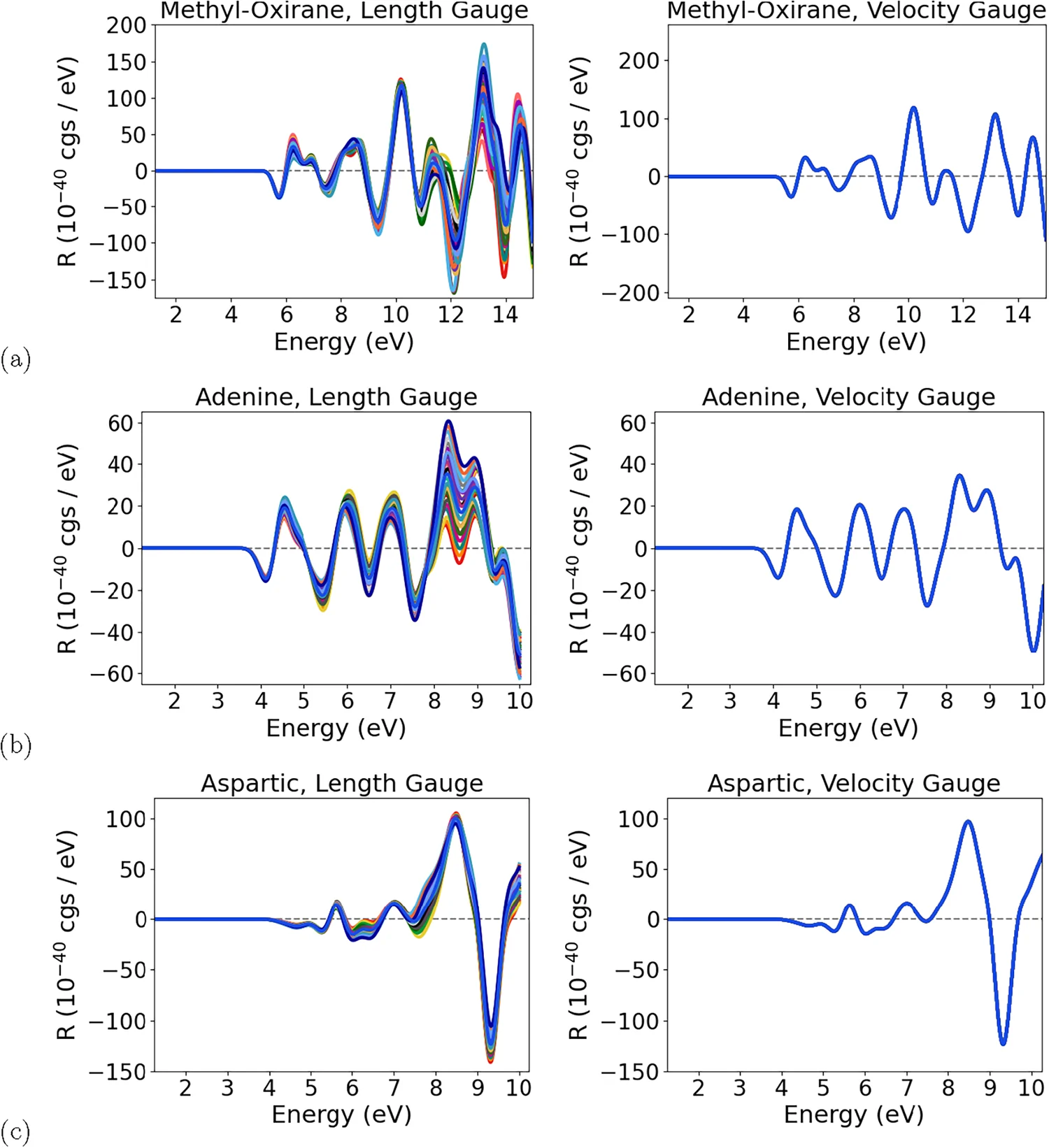
Molecular ECD spectra, for (a) methyl-oxirane, (b) adenine, and (c) aspartic, calculated within length gauge (left column) and velocity gauge (right column). The titles of the plots give the used gauge. The spectra were calculated for 27 calculation origins, lying within a 20 Å cube, using augmented double-ζ polarized basis sets.

In the length gauge, peak intensities vary considerably for different origins, with deviations reaching up to ∼122 *R*·10^–40^ cgs/eV for methyl-oxirane’s spectra around 12 eV. Sometimes also the sign of the peak intensity can change. For the velocity gauge, all spectra overlap and we observe no origin dependence.

Figure 5 shows the comparison of length and velocity gauge ECD spectra across 27 different origins for the protein crambin, calculated using the dzp basis sets. We observe the same behavior as for the small molecules: we obtain a strong origin dependence in the length gauge (Figure 5, left column) while this is not the case for the spectra calculated in the velocity gauge (Figure 5, right column).

**Figure 5:**
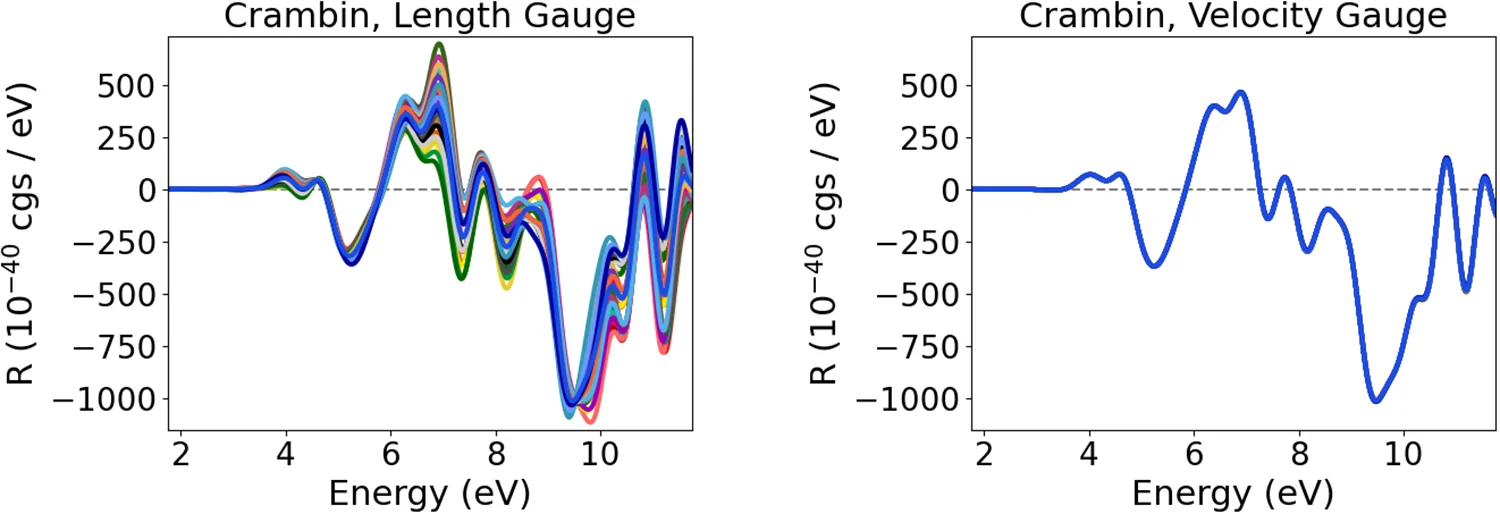
Crambin’s ECD spectra calculated within the length gauge (left) and the velocity gauge (right). The spectra were calculated for 27 calculation origins, lying within a 20 Å cube, using double-ζ polarized basis sets.

Figure 6 compares the ECD spectra of cytosine (upper panel) and cytosine on Ag_147_ (lower panel). Comparing the length gauge results from cytosine (upper panel of Figure 6, left column) and the hybrid system (lower panel of Figure 6, left column), we witness an amplification of the origin dependence for the hybrid system. As for the small molecules, length gauge results for the hybrid system show a significant origin dependence with peaks changing sign (see the spectrum close to 6.9 eV for example). For the hybrid system as well, the velocity gauge remains origin-independent.

**Figure 6:**
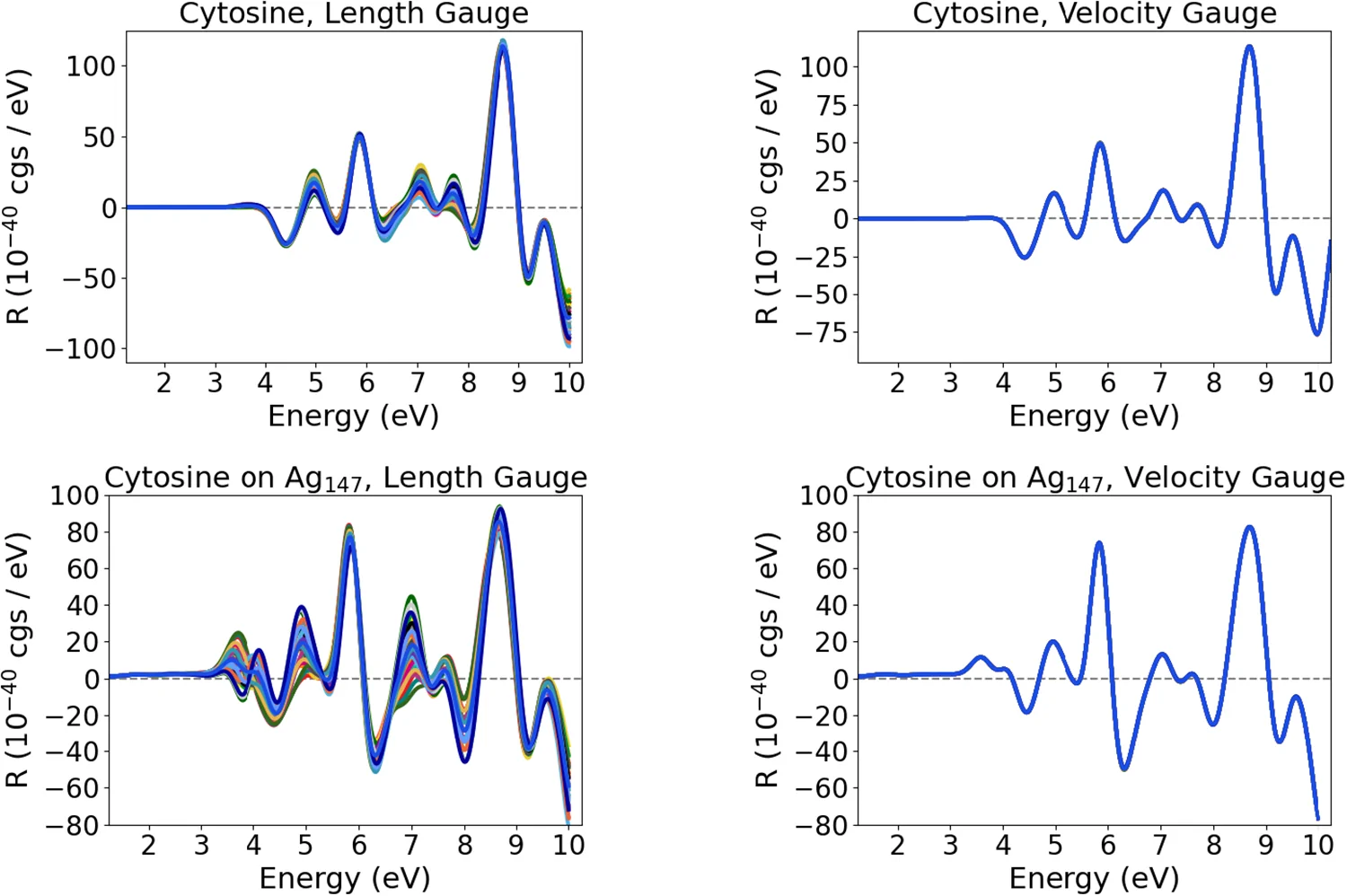
ECD spectra from cytosine (upper panel) and cytosine on Ag_147_ (lower panel) calculated within the length gauge (left) and velocity gauge (right). The spectra were calculated for 27 calculation origins, lying within a 20 Å cube, using augmented double-ζ polarized basis sets.

**Figure 7:**
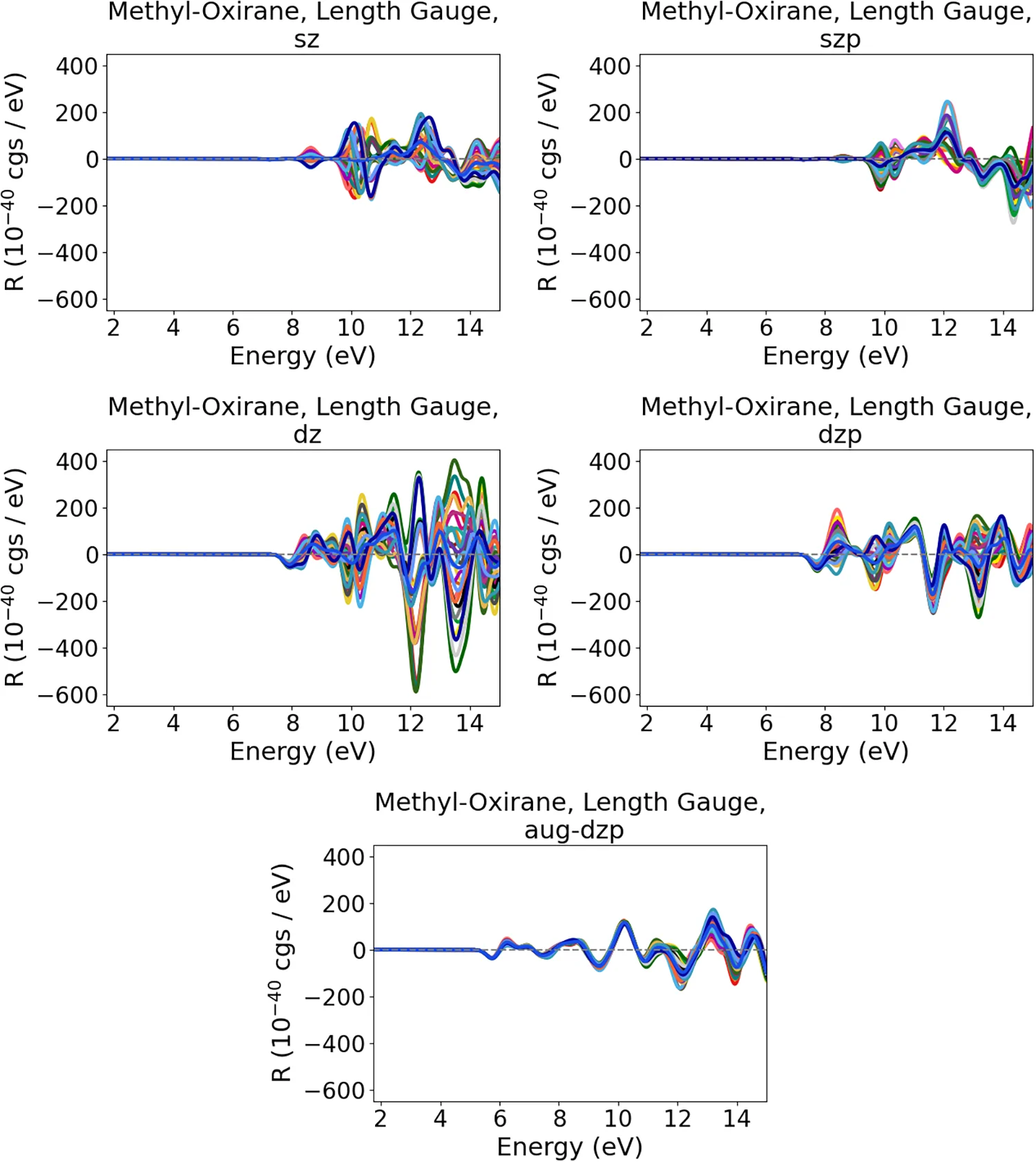
ECD spectra calculated in the length gauge for different basis set qualities. The used single-ζ (sz), single-ζ polarized (szp), double-ζ (dz), double-ζ polarized (dzp), and augmented double-ζ polarized (aug-dzp) basis set qualities are indicated in the titles. The spectra were calculated for 27 calculation origins, lying within a 20 Å cube.

To verify the internal consistency of our implementation, we analyzed how the origin dependence in methyl-oxirane’s ECD spectra evolves as the basis-set size is systematically increased from single-ζ (sz) to aug-dzp quality (Figures 7 and S3 in the SI). As before, the length gauge results exhibit pronounced origin dependence, particularly for basis sets ranging from sz to dzp, whereas the velocity gauge calculations show no noticeable origin dependence across all basis-set sizes. For the length-gauge results, the origin dependence reduces for larger basis sets. For aug-dzp below the ionization threshold the ECD spectra start to converge.

**Figure 8:**
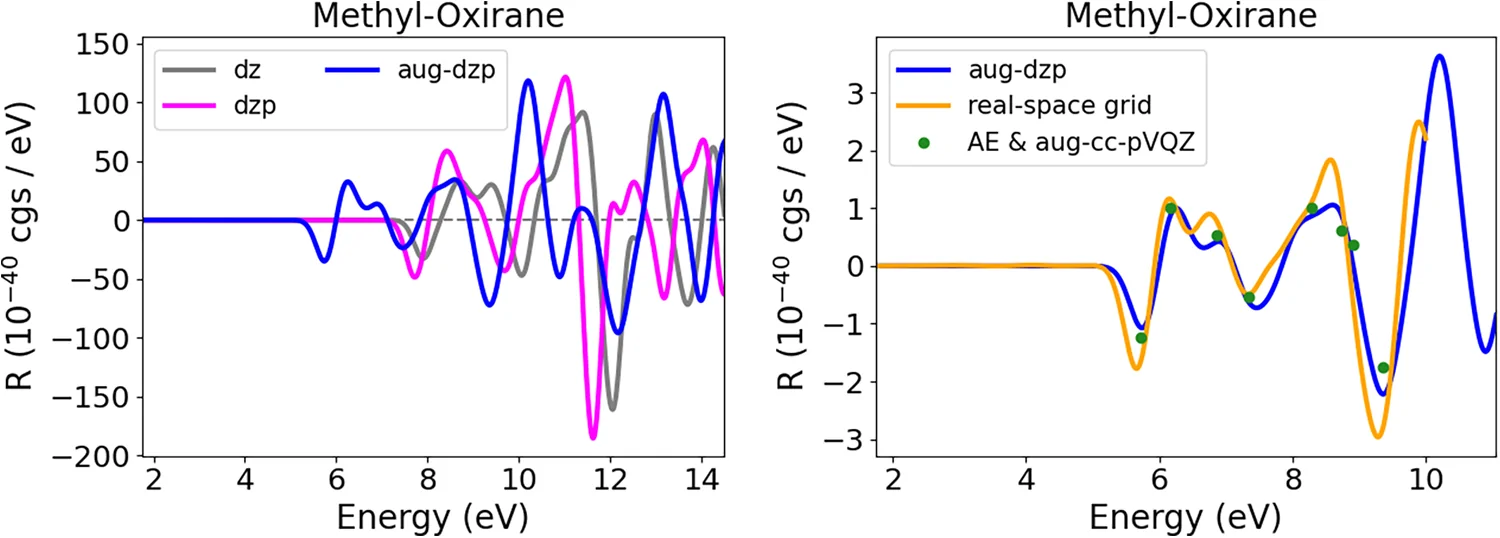
Left panel: Methyl-oxirane’s ECD spectra calculated in the velocity gauge, using double dz, dzp, and aug-dzp basis set qualities, that are indicated in the legend. Right panel: Velocity-gauge ECD spectrum of methyl-oxirane (aug-dzp) compared to real-space grid results and literature feature positions from ref 34, Figure 7, left-part, calculated using all-electron (AE) basis sets and quadruple-ζ polarized basis sets (aug-cc-pVQZ), as well.

**Figure 9:**
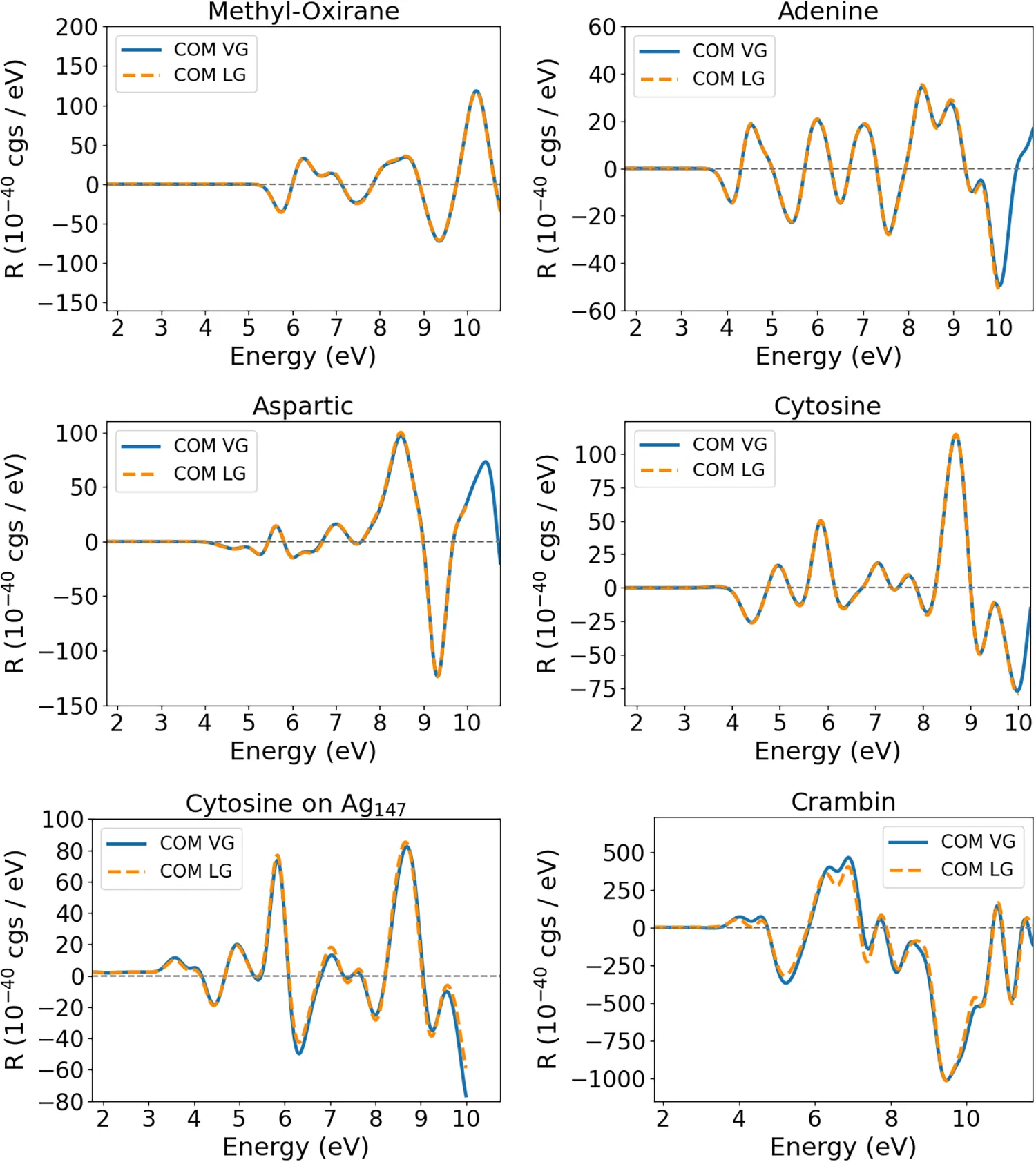
ECD spectra of studied systems, calculated using the center-of-mass as the origin. The spectra were calculated in velocity gauge (blue lines) and in length gauge (dashed-orange lines).

Examining the velocity gauge spectra, it becomes apparent that the positions of spectral features shift significantly when increasing the basis set size from smaller sets to aug-dzp quality, see Figure S3. This motivated a comparison of velocity-gauge results obtained with different double-ζ basis sets in the left part of Figure 8. In particular, the peak positions of the aug-dzp spectrum differ significantly from those of the dz and dzp spectra (see left part of Figure 8). The right part of Figure 8 shows the comparison of ECD spectra to spectra obtained with real-space grid calculations and to feature positions from literature (ref 34), that were computed using all-electron (AE) and augmented quadruple-ζ polarized (aug-cc-pVQZ) basis sets. Here, all shown spectra are normalized to the first peak, which is located in the vicinity of 6 eV. The positions of features from the velocity-gauge spectrum, real-space grid spectrum, and from literature are overall in good agreement. The feature comparison is revisited in the following section.

In Figure 9 we compare velocity-gauge ECD spectra for the molecules and the smaller hybrid system to length-gauge calculations for the center-of-mass (COM) origin. The COM length-gauge spectra agree closely with the velocity-gauge spectra for the systems.

Having tested internal consistency of our implementation, we tested the practical applicability of RT-TDDFT CD calculations with LCAO basis sets in the large-system regime. Here we show the results from our code for a hybrid system consisting of methyl-oxirane on Au_923_ (see lower part of Figure 3). Comparison of the ECD spectrum of the hybrid system with that of the isolated molecule (Figure 10) reveals two effects arising from the molecule–nanoparticle interaction. First, a shift of the resonances is observed: features below 10 eV appear predominantly blue-shifted, whereas those above 10 eV are red-shifted. In this comparison, the broad and sharp resonances of the hybrid system (solid orange line) in the energy range from ∼7.7 to 9.4 eV are assigned to the broader resonance of methyl-oxirane (dashed blue line) spanning ∼7.8 to 8.9 eV. Importantly, we can also identify damping and enhancement of shifted molecular resonances. At this, methyl-oxirane’s resonances between ∼6.0 eV and ∼9.7 eV are damped while the first resonance and the resonance in the vicinity of 10 eV are enhanced. For the RT-TDDFT calculations, each delta-kick direction required approximately 39 h of wall-clock time under not fully optimized computational settings.

**Figure 10:**
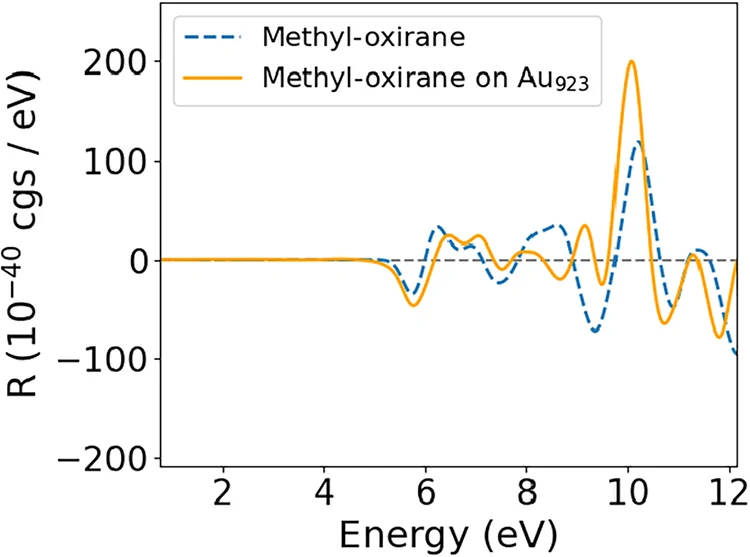
ECD spectra of methyl-oxirane (dashed blue line) and methyl-oxirane on Au_923_ (solid orange line). The calculations were carried out using the velocity gauge.

## Discussion

Our length-gauge calculations for large molecules and hybrid systems exhibit pronounced origin dependence (see Figure 5, left column and Figure 6, lower panel, left column), which is most probably related to their extension and heterogeneity. The system’s spatial separation between subunits or subsystems can exacerbate the impact of the chosen origin, due to the explicit involvement of the position operator in the length gauge (see eq 4), leading to discrepancies severely complicating a meaningful interpretation of spectral features.

We observed that the origin dependence is more pronounced near or above the ionization threshold. This could be related to deficiencies of the LCAO basis sets in the description of continuum states.^15,37^

In contrast to the length gauge, the velocity gauge calculations are effectively free of the origin dependence and produce the same spectrum irrespective of the origin (see Figure 5, right column and Figure 6, right column). While these results are very promising, we do not claim a complete resolution of the origin dependence. We anticipate, for example, that higher-rung exchange–correlation functionals, such as hybrid functionals, introduce additional orbital and nonlocal dependencies that can increase the sensitivity of real-time response calculations to origin-dependence in LCAO implementations. Moreover, we especially expected that origin effects may still arise for hybrid systems or large molecules, where the Coulomb field of one subsystem acts as an external potential for another. However, in our current tests of the velocity gauge implementation, we do not observe any significant origin dependence even for large molecules or hybrid systems. Representative examples can be seen in the right columns of Figures 5 and 6 (lower panel) and were discussed above.

We also investigate the COM-coordinate spectra in the length and velocity gauge. COM is commonly used in the length gauge and considered to be reliable.^20,31,35^ As shown in Figure 9, the COM spectra obtained in the length and velocity gauges agree closely across all investigated systems. While deviations between velocity- and length-gauge COM ECD spectra are more pronounced for hybrid systems and large molecules, they still remain negligible. These results suggest that COM can serve as an alternative for mitigating origin-dependent effects when a velocity-gauge implementation is not available; however, caution is advised—especially for extended systems as hybrid systems, and large molecules.

During our investigation of how basis-set size affects origin dependence, we observed substantial shifts in the positions of methyl-oxirane’s spectral features when increasing the basis quality from sz to dzp to aug-dzp, with aug-dzp differing most from the others. This led us to compare the feature positions in the velocity-gauge aug-dzp ECD spectrum with those from real-space grid calculations and from ref 34. All our spectra were Gaussian-broadened with a width of 0.2 eV, which smooths individual features (see Computational Details section). The dominant features in the methyl-oxirane velocity-gauge aug-dzp ECD spectrum (Figure 8) agree well with both the real-space grid results and the AE/aug-cc-pVQZ data in Figure 7 of ref 34. This shows that aug-dzp provides an adequate description of the ECD spectrum of methyl-oxirane below the ionization threshold, in line with previous findings that aug-dzp is sufficient for ECD calculations.^30^ While the results from Figures 7 and S3 indicate convergence of the length gauge toward velocity gauge results in the complete basis set limit, achieving this with length gauge would require basis sets that are prohibitively large for large molecules and realistically sized hybrid systems. The results presented confirm the excellent performance of our velocity gauge implementation within the PAW formalism for LCAO basis sets of moderate size.

Furthermore, we demonstrate the applicability of the velocity gauge RT-TDDFT ECD implementation to large-scale systems by calculating the ECD spectrum of a large molecule–nanoparticle hybrid system (see Figure 10 and its discussion). Our calculations demonstrate the scalability of the implementation for complex molecular–nanoparticle hybrids. These results open the possibility of predicting ECD spectra for large hybrid systems and proteins.

## Conclusion

In this study, we aimed to achieve origin-independent CD results using LCAO basis sets for RT-TDDFT in the PAW formalism, implemented in GPAW. We identified the explicit coordinate dependence of the time-dependent KS-Hamiltonian in the length gauge as the primary source of error leading to origin dependence. Our GPAW RT-TDDFT velocity gauge implementation eliminates this error and enables origin-independent results from LCAO calculations within the PAW formalism. Our implementation allows us to obtain origin-independent results using moderately sized basis sets, regardless of the size or complexity of the systems under study.

For completeness, we note that large and flexible systems may require additional modeling beyond RT-TDDFT calculations. Several conformers and vibrational contributions associated with a finite temperature, for example, can affect the ECD spectrum. These effects can be addressed by combining GPAW RT-TDDFT calculations with external conformer-search, which can be accelerated using machine leaning codes such as Bayesian Optimization Structure Search,^19,50^ and by averaging spectra over molecular configurations obtained from finite-temperature molecular dynamics for example. Although this increases the number of required RT-TDDFT simulations, the calculations for different conformers or snapshots are independent and can therefore be efficiently distributed on HPC resources. Moreover, RT-TDDFT remains advantageous for systems with a high density of electronic states, since the full spectral response is obtained from a single time propagation. Further speedups on large systems may be achieved by using extrapolation schemes as those introduced in ref 21,23 , which is a promising direction for future GPAW developments.

Overall, without the need for excessively large basis sets or origin-specific corrections, ECD spectra for large systems exceeding 1000 atoms are now within reach.

## Supplementary Material



## Notes

Basis set generation; atomic structure of studied systems; coordinates of shifted origins; velocity gauge ECD spectra from different basis set qualities (PDF)
