# Supplementary material for: Origin-Independent Circular Dichroism Spectra for Molecular and Hybrid Systems with Real-Time Time-Dependent Density Functional Theory
Source: J Phys Chem A. 2026 Oct 20;130(44):8960–71. doi: 10.1021/acs.jpca.6c03456 (PMC13746803; doi:10.1021/acs.jpca.6c03456)
Supplement: Supplementary file 1 [file jp6c03456_si_001.pdf]

Supporting Information

Origin-Independent Circular Dichroism Spectra for Molecular and Hybrid Systems with Real-Time Time-Dependent Density Functional Theory

Maria J. Weseloh,[*,†] Mikael Kuisma,[‡] Xi Chen,[¶] and Patrick Rinke[§,†,∥,⊥]

†*Department of Applied Physics, Aalto University, P.O.Box 11100, 00076 AALTO, Finland*

‡*CAMD, Department of Physics, Technical University of Denmark, 2800 Kgs. Lyngby, Denmark*

¶*Lanzhou Center for Theoretical Physics, Lanzhou University, China*

§*Physics Department, TUM School of Natural Sciences, Technical University of Munich, Garching, Germany*

∥*Atomistic Modelling Center, Munich Data Science Institute, Technical University of Munich, Walther-Von-Dyck Str. 10, 85748 Garching, Germany*

⊥*Munich Center for Machine Learning (MCML), Munich, Germany*

E-mail: maria.weseloh@aalto.fi

Basis set generation

We augmented GPAW's dzp basis sets with numerical Gaussian-type orbitals (NGTOs) using GPAW's built-in function `generate_nao_ngto_basis`, as shown in the following code and in our GPAW documentation *:

```
from gpaw.lcao.generate_ngto_augmented import \
    create_GTO_dictionary as GTO, generate_nao_ngto_basis

gtos_atom = {'H': [GTO('s', 0.02974),
                   GTO('p', 0.14100)],
             'C': [GTO('s', 0.04690),
                   GTO('p', 0.04041),
                   GTO('d', 0.15100)],
             'N': [GTO('s', 0.06124),
                   GTO('p', 0.05611),
                   GTO('d', 0.23000)],
             'O': [GTO('s', 0.07896),
                   GTO('p', 0.06856),
                   GTO('d', 0.33200)],
             }

for atom in ['H', 'C', 'O']:
    generate_nao_ngto_basis(atom, xc='PBE', name='aug',
                            nao='dzp', gtos=gtos_atom[atom])
```

Figure S1: Python script used for augmented basis-set generation.

The NGTOs' parameters correspond to the diffuse functions of the elements' aug-cc-pvdz basis sets from Basis Set Exchange †. We visually inspected the augmenting NGTO functions generated with GPAW's `gpaw-analyse-basis`. As can be seen in Figure S2, the NGTOs exhibit smooth radial behavior, consistent spatial ordering, and no indications of unphysical exponents. Across H, C, N, and O, the NGTO shapes follow the expected trends and appear physically plausible. The aug-dzp basis sets from this study are available for download at `https://doi.org/10.5281/zenodo.19854586`.

* `https://gpaw.readthedocs.io/tutorialsexercises/opticalresponse/circular_dichroism/rttddft.html\#lcao-mode`

† https://www.basissetexchange.org/

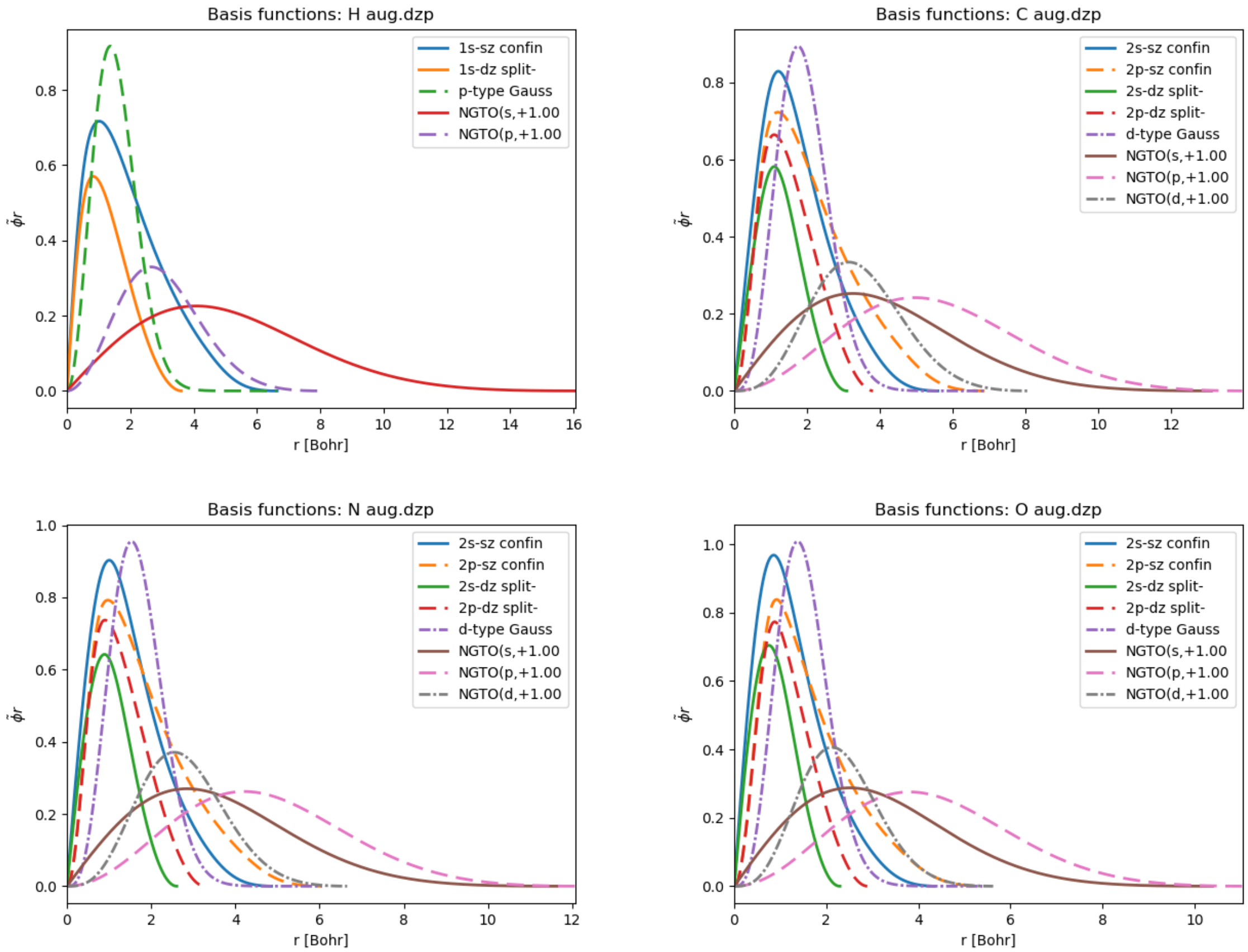

Figure S2: Radial basis functions for H, C, N, and O in the aug-dzp basis. The plots show the dzp valence functions together with the NGTO augmenting functions. The legend distinguishes confined sz functions, split-valence dz functions, Gaussian p- or d-type polarization functions, and the NGTO augmenting functions. The radial coordinate r is given in Bohr, and $\tilde{\phi}r$ denotes the plotted radial functions.

Atomic structure of studied systems

The Cartesian coordinates of the structures used in this work are available as XYZ files at `https://doi.org/10.5281/zenodo.20393303`.

Coordinates of the shifted origins

Table S1: Origin shifts used in for origin-dependent calculations. The shifts span a cube of side length 20 Å with coordinates $x, y, z \in \{-10, 0, 10\}$ Å.

x (Å)	y (Å)	z (Å)
-10	-10	-10
-10	-10	0
-10	-10	10
-10	0	-10
-10	0	0
-10	0	10
-10	10	-10
-10	10	0
-10	10	10
0	-10	-10
0	-10	0
0	-10	10
0	0	-10
0	0	0
0	0	10
0	10	-10
0	10	0
0	10	10
10	-10	-10
10	-10	0
10	-10	10
10	0	-10
10	0	0
10	0	10
10	10	-10
10	10	0
10	10	10

Velocity gauge ECD spectra from different basis set qualities

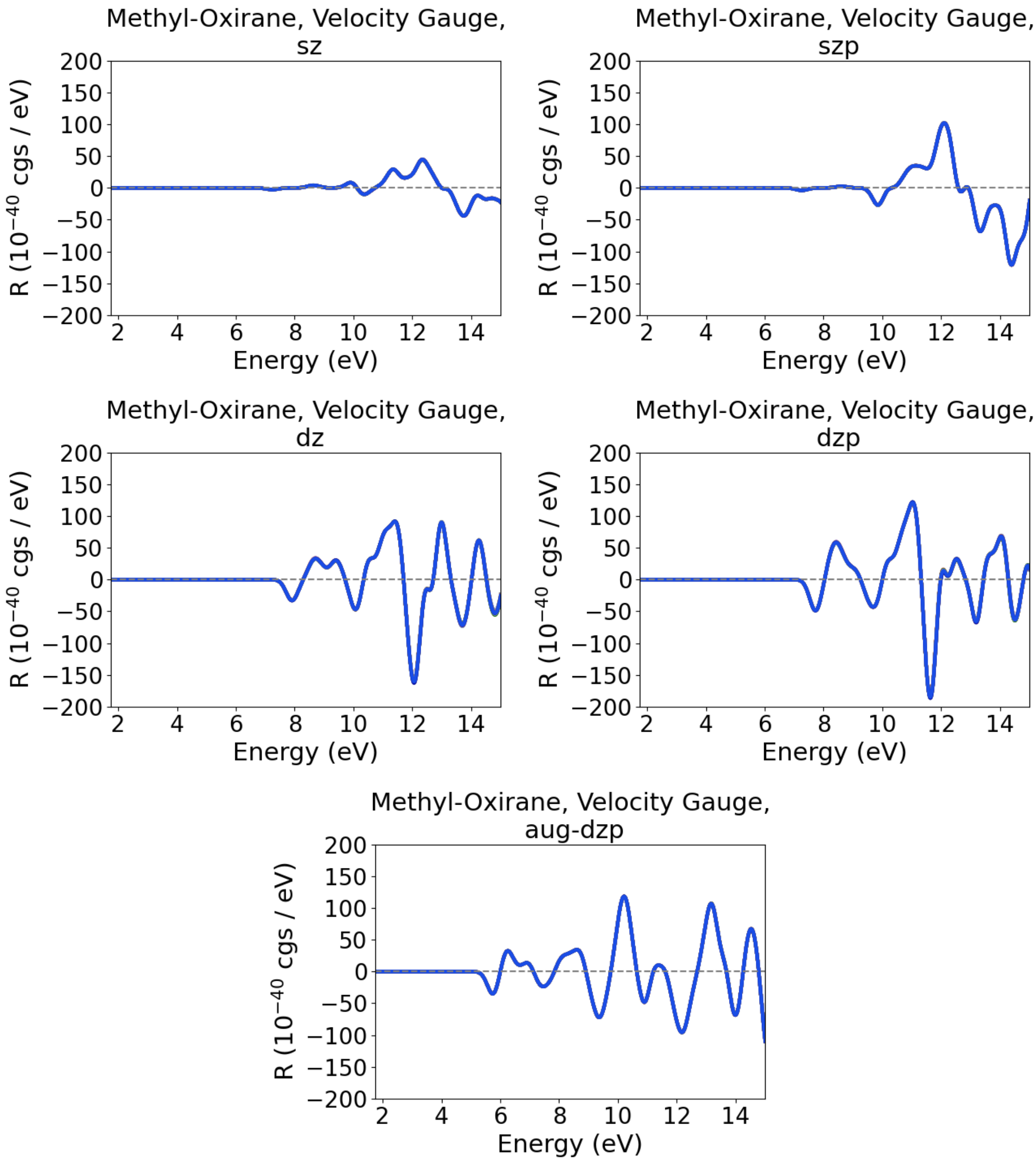

Figure S3: ECD spectra calculated in the velocity gauge for different basis set qualities. The used sz, szp, dz, dzp, and aug-dzp basis set qualities are indicated in the titles. The spectra were calculated for 27 calculation origins, lying within a 20 Å cube. The last plot shows all ECD calculated with dz-type basis set together. Here, only the COM-spectra were plotted, which agree with the same ECD spectra from other origins.
